# Convex Non-Negative Matrix Factorization for Brain Tumor Delimitation from MRSI Data

**DOI:** 10.1371/journal.pone.0047824

**Published:** 2012-10-23

**Authors:** Sandra Ortega-Martorell, Paulo J. G. Lisboa, Alfredo Vellido, Rui V. Simões, Martí Pumarola, Margarida Julià-Sapé, Carles Arús

**Affiliations:** 1 Departament de Bioquímica i Biología Molecular, Universitat Autònoma de Barcelona (UAB), Cerdanyola del Vallès, Spain; 2 Centro de Investigación Biomédica en Red en Bioingeniería, Biomateriales y Nanomedicina (CIBER-BBN), Cerdanyola del Vallès, Spain; 3 Institut de Biotecnologia i de Biomedicina, Universitat Autònoma de Barcelona (UAB), Cerdanyola del Vallès, Spain; 4 Department of Mathematics and Statistics, Liverpool John Moores University (LJMU), Liverpool, United Kingdom; 5 Department of Computer Languages and Systems, Universitat Politècnica de Catalunya (UPC), Barcelona, Spain; 6 Department of Medical Physics, Memorial Sloan-Kettering Cancer Center, New York, New York, United States of America; 7 Murine Pathology Unit, Centre de Biotecnologia Animal i Teràpia Gènica, Departament de Medicina i Cirurgia Animals, Universitat Autònoma de Barcelona (UAB), Cerdanyola del Vallès, Spain; Instituto de Investigación Sanitaria INCLIVA, Spain

## Abstract

**Background:**

Pattern Recognition techniques can provide invaluable insights in the field of neuro-oncology. This is because the clinical analysis of brain tumors requires the use of non-invasive methods that generate complex data in electronic format. Magnetic Resonance (MR), in the modalities of spectroscopy (MRS) and spectroscopic imaging (MRSI), has been widely applied to this purpose. The heterogeneity of the tissue in the brain volumes analyzed by MR remains a challenge in terms of pathological area delimitation.

**Methodology/Principal Findings:**

A pre-clinical study was carried out using seven brain tumor-bearing mice. Imaging and spectroscopy information was acquired from the brain tissue. A methodology is proposed to extract tissue type-specific sources from these signals by applying Convex Non-negative Matrix Factorization (Convex-NMF). Its suitability for the delimitation of pathological brain area from MRSI is experimentally confirmed by comparing the images obtained with its application to selected target regions, and to the gold standard of registered histopathology data. The former showed good accuracy for the solid tumor region (proliferation index (PI)>30%). The latter yielded (i) high sensitivity and specificity in most cases, (ii) acquisition conditions for safe thresholds in tumor and non-tumor regions (PI>30% for solid tumoral region; ≤5% for non-tumor), and (iii) fairly good results when borderline pixels were considered.

**Conclusions/Significance:**

The unsupervised nature of Convex-NMF, which does not use prior information regarding the tumor area for its delimitation, places this approach one step ahead of classical label-requiring supervised methods for discrimination between tissue types, minimizing the negative effect of using mislabeled voxels. Convex-NMF also relaxes the non-negativity constraints on the observed data, which allows for a natural representation of the MRSI signal. This should help radiologists to accurately tackle one of the main sources of uncertainty in the clinical management of brain tumors, which is the difficulty of appropriately delimiting the pathological area.

## Introduction

Nuclear magnetic resonance (MR) is a key technique for the non-invasive analysis of brain tumors in the field of neuro-oncology. The spectroscopic variant of MR, Magnetic Resonance Spectroscopy (MRS), provides radiologists with a precise metabolic signature of the target tissue, allowing the identification of a wide array of molecules that may be present in tissues, even at low concentration (mM range).

Magnetic Resonance Spectroscopic Imaging (MRSI) combines both spectroscopic and imaging acquisition modalities to produce spatially localized spectra, and thus delivers information about the spatial localization of molecules. This modality has been successfully applied to monitoring the metabolic heterogeneity of human brain tumors [1]–[4].

The rich information contained in MR signals makes them ideally suited to the application of pattern recognition (PR) techniques [5], [6]. Over the last two decades, these techniques have been successfully applied to the problem of knowledge extraction from human brain tumor data, for diagnosis and prognosis of different pathologies, mostly using single-voxel proton MRS (SV ^1^H-MRS) [7]–[12].

Even when substantial advances have been achieved in the application of PR to the problems of brain tumor type and grade discrimination, a “gray zone” of uncertainty in tissue characterization still remains, where spectra of different tissue types mix. To address this limitation, methods that provide accurate discrimination of tissue types from the MR spectra, with support from MR images, would be required, ideally without the need for prior information regarding tumor type and grade. This, from the PR viewpoint, is an unsupervised modeling task.

**Figure 1:**
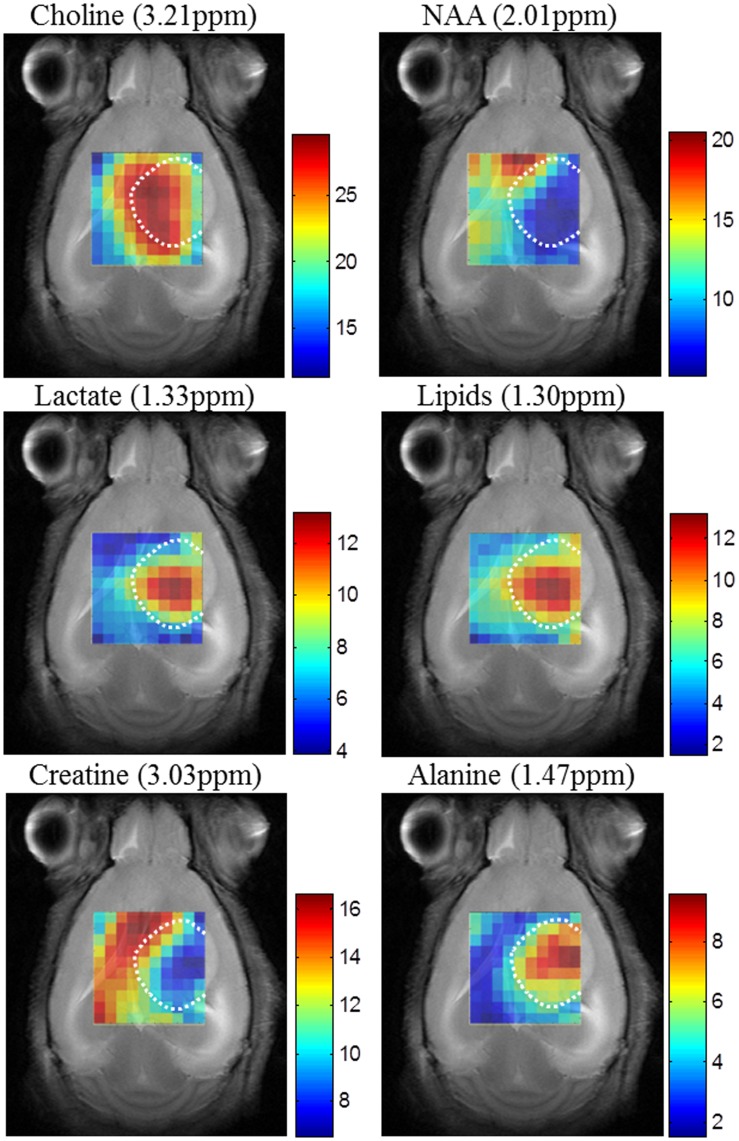
Peak maps of principal metabolites for mouse C69, harboring a glioblastoma. It was scanned at 7 Tesla by PRESS-MRSI with 136 ms echo-time. MRSI data were acquired with Bruker ParaVision 4.0, and Fourier interpolated to 32×32 voxels, with a final PRESS MRSI data grid of 10×10 voxels. Line broadening adjustments and zero order phase correction were carried out. Then, the data were fed into a home-built module for MRSI post-processing, where the 4.5–0 ppm region of each spectrum was individually normalized to Unit Length (UL2). Each map shows the peak height in absolute values of the studied metabolite in each voxel. The white dotted lines highlight the tumoral mass according to T2-W MR images. The scales reflected in the colors coding are in arbitrary units.

As an example of the need for such methods, for instance for the problem of discriminating normal from abnormal tissue, figure 1 illustrates that no single metabolite image produces, by itself, a consistent segmentation. This figure compiles six 10×10 color-coded maps displaying the spatial accumulation of the main metabolites detected by MRSI in a mouse model of brain tumor (choline, N-acetyl aspartate (NAA), lactate, lipids, creatine, and alanine), superimposed over the T2 weighted (T2-W) MR image [13]. More sophisticated approaches for metabolite imaging have been proposed, for example using selected metabolite concentration estimates and exploiting spatial information to improve tissue heterogeneity definition [14], but, as in figure 1, fully consistent segmentation using a single metabolite concentration does not seem evident and varies in quality with tumor type in the three patients investigated.

In the past, the problem has been mostly undertaken from a supervised point of view, through the so-called nosologic imaging approach, in which an image obtained with PR is color-coded according to its histopathological class [3], [15] or according to an index of “metabolic abnormality” above a certain threshold, e.g. the choline-containing compound - NAA index (CNI) [4], [16].

In the current study, we approach this problem using signal source extraction techniques. By considering spectra from a grid of voxels (a small cubic based volume element (voxel) for the region to be sampled with MRS) in a region of the brain (also known as volume of interest, VOI), we aim to separate the constituent source signals on the assumption that they are mixed linearly in each single-voxel spectral measurement. This is a fair assumption, given that *in vivo* spectroscopy signals are the result of overlapping peaks, caused by broad resonances [17] as opposed to narrow peaks that are characteristic of high-resolution spectra of compounds in solution. Through source extraction, if there were different constituent tissue types present in these heterogeneous areas of the brain they might be separately identified and quantified. As a result, the level of tissue type (class) assignment for the sources of each voxel spectrum could also be quantified. This provides us with an unsupervised class assignment alternative to the standard supervised classification of a complete spectrum.

Importantly, this methodology does not involve combining spectra from different subjects, thus focusing on intra-subject variation without contamination from inter-subject overlaps.

We propose the use of an unsupervised method for matrix factorization, specifically the convex [18] variant of Non-negative Matrix Factorization (NMF, [19], [20]), for the extraction of the sources underlying the MRS signal, the identification of tumor type-specific sources, and the generation of image maps providing an adequate delimitation of the pathological area.

Different variants of NMF have previously been applied in the context of neuro-oncology to distinguish normal from abnormal masses, such as the one proposed by Lee and Seung [20], used in [21], and the constraint NMF (cNMF) technique used in [22] and [23]. Unlike the Convex-NMF technique [18] used in our study, both variants require the source and mixing matrices to be non-negative. This is an important advantage of Convex-NMF, given that the analyzed MRSI data can take negative values. Previous attempts of using NMF on similar data have resorted to either extra-long echo time (TE) spectra at 280 ms [23], or magnitude spectra [21], both of which render only positive peaks.

In [21], authors decomposed the observed spectra of multiple voxels into what they called abundance distributions and constituent spectra. The accuracy of the estimated abundances was validated on phantom data, i.e. synthetic samples of known composition, while the extracted spectra were validated with their correlation with MRS data from 20 patients, on the choline and NAA peak areas. In [22], synthetic and real MRS data were used to calculate the error between the extracted sources and the observed spectra; and in [23], *in vivo* MRS and MRI data were used to evaluate the results.

For the current paper, a pre-clinical study was carried out using brain tumor-bearing mice. *In vivo* MRI and MRS were acquired from the brain and tumor tissue, along with *ex vivo postmortem* histology slides of the same animals. This enabled us not only to evaluate the correlation of the sources obtained with the observed spectra, but also to evaluate how accurate the calculated maps were in delineating the tumor region, with respect to a true biological correlate acquired *ex vivo*, upon sacrifice of the animal. This *ex vivo* – *in vivo* correlation is virtually impossible to achieve with human brain tumors.

In summary, the aims of the experiments carried out for this study were to: 1) Explore the comparative ability of NMF methods to extract the constituent sources of MRSI data; 2) Generate tissue labels in a fully unsupervised mode; and 3) Investigate the possibility of creating an accurate delimitation of the tumor area, after identifying the sources that describe the tumor tissue. This technique is unsupervised in the sense that labels (tumor or normal tissue) are not required to create a model of the analyzed MRSI data, i.e. to find the MRS sources. This is important since routine histopathological assignment of the class and grade of specific tumors has been shown not to be fully reproducible [24]–[26] and may introduce unwanted variation in the supervised analysis.

## Materials and Methods

### Ethics Statement

All studies were approved by the local ethics committee (*Comissió d’Ètica en l’Experimentació Animal i Humana* (CEEAH). Available: http://www.recerca.uab.es/ceeah. Accessed 17 May 2012.), according to the regional and state legislation (protocol DARP-3255/CEEAH-530). Mice were obtained from Charles River Laboratories (France) and housed at the animal facility of the *Universitat Autònoma de Barcelona* (*Servei d’Estabulari*) [27].

### Materials: Description of the Data

Glioblastoma multiforme (GBM) is a malignant intra-cranial tumor, of high incidence and very poor prognosis in humans. Seven GL261 GBM-bearing mice [27] were scanned at 7 Tesla by PRESS-MRSI, using two different echo times: short, 12 ms (STE); and long, 136 ms (LTE). Compared to LTE, STE spectra typically show more complex patterns, including signals from metabolites with short and long T2 relaxation times. In LTE spectra, signals with short T2 will be lost, such as most lipids and macromolecules, remaining visible only spins with longer T2, i.e. small metabolites. Thus, the lactate methyl signals resonating close to the lipid methyl groups become more visible in LTE spectra. In addition, due to J-coupling effects, the methyl protons of alanine and lactate will appear inverted in LTE spectra, that is, they will undergo a 180 degree phase shift compared to other visible metabolites [15].

Six of these mice, namely C69, C71, C32, C179, C233, and C234, were described in [27]. A seventh mouse, namely C278, was used in this work to increase the number of experiments. This animal was handled (tumor induction) and scanned exactly as reported for the other six. The PRESS MRSI data grid was formed by an array (10×10 voxels) with an in-plane resolution of 0.55×0.55 mm and a 1 mm slice thickness in the 3^rd^ dimension [27]. This volume of interest was manually positioned approximately in the center of the brain, based on the reference image, in a way that it would include most of the tumor mass and also part of the normal/peritumoral brain parenchyma.

Sources extracted as described later on in this section were used to create maps delimiting the tumor region. These maps were compared to the gold standard, which, in this study, is Ki67 stained sections of the post-mortem, paraffin-embedded mice brains as in [28].

Ki67 immunostaining allows the spatial determination of the proliferating population of cells in an individual tumor [29]. The proliferation index (PI), also called mitotic index, was calculated for each tumor by, first, immunohistochemical technique on paraffin-embedded slices with the Ki-67 antibody (BD Biosciences Pharmingen 550609) and, second, subsequently counting the number of positive cells per field at 40×magnification. This was achieved with the Definiens Developer XD 1.2 software (Definiens website. Available: http://www.definiens.com. Accessed 17 May 2012.) “Image Registration: MRI & Tissue Slide” package, which correlates the MRI with the corresponding Ki-67 immunostained slice through an affine transformation. The Ki67 immunostained slice that best matched the MRI slice was chosen by the pathologist (co-author, M. Pumarola) following anatomical criteria. In this particular murine model, and according to the pathologist, PI>30% would correspond to a safe threshold for the solid tumoral region, whereas a PI≤5% would correspond to definitely non-tumor (excluding reactive gliosis and other phenomena), in agreement with similar studies (PI<12% for control) [30]. In humans, the PI values for glioblastomas vary considerably, reflecting, to some extent, tumor heterogeneity [29], [31].

An additional way of validating the obtained sources makes use of the labeling procedure described in [32], to compare the sources obtained with the mean spectra of tumor and non-tumor regions, similarly to the Ki-67 threshold validation described above. In [32], subsets of tumoral and non-tumoral regions were labeled, for each of the investigated mice, according to the following criteria: first, the spectra should not correspond to voxels at the edge of the PRESS-VOI, where signal to noise ratio (SNR) tends to be lower; and second, as in [33], they had not been collected over, or close to, the tumor borderline, to avoid as much as possible voxel ‘bleeding’ between tumor/non-tumor regions.

### Non-negative Matrix Factorization for Source Extraction

In NMF methods [19], [20], the data matrix 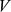 (of dimensions 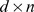, where 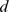 is the data dimensionality and *n* is the number of observations), is approximately factorized into two non-negative matrices, the matrix of sources or data basis 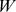 (of dimensions 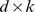, where 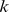 is the number of sources, and 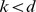) and the mixing matrix 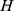 (of dimensions 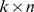, each of whose columns provides the encoding of a data point: the spectrum of a voxel in this study). The product of these two matrices provides a good approximation to the original data matrix, in the form:
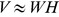
(1)


In this study, the following NMF methods, which cover a wide palette of algorithmic alternatives, were considered:

- Euclidean distance update algorithm (herein referred to as *euc*) [20]. The objective function is optimized with multiplicative update rules for *W* and *H*:

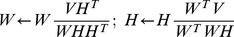



Monotonic convergence of the algorithm can be proven [20]. These update equations preserve the nonnegativity of 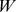
and 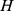, and constrain the columns of 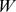 to sum to unity.

- Alternating least squares (*als*) [19]. This technique alternately fixes one matrix and updates the other.

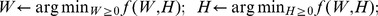
where 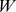
and 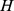 are updated as follows:



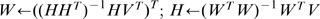



setting all negative elements in 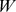
and 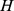 to zero.

- Alternating non-negative least squares using projected gradients (*alspg*) [34]. The equations for 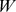
and 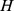 in the alternating least squares method above are solved here using projected gradients. For 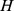 this entails:

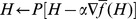
; where 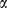 is the step size; and 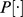 is a bounding function that ensures that the solution remains within the boundaries of feasibility. The gradient function is computed as 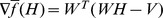. The same approach is used to calculate 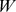.
- Alternating least squares with Optimal Brain Surgeon (OBS) [35] (*alsobs*) [36]. Similar to alternating least squares, this algorithm alternately solves the least squares equations for 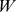
and 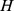. The negative elements in 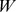
and 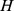 are set to zero and the rest are adjusted using the OBS method, through second-order derivatives. The update rules for 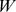
and 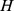 are:

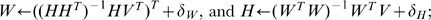



where 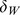 and 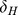 act as regularization terms and are responsible for eliminating the less important elements of 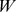
and 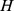, respectively (the original OBS was used as a weight pruning mechanism in artificial neural networks), thus re-adjusting the remaining elements optimally. More implementation details can be found in [37].

- Convex-NMF [18]. To achieve interpretability, this method imposes a constraint that the vectors (columns) defining 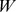 must lie within the column space of 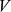, i.e. 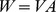 (where 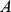 is an auxiliary adaptive weight matrix that fully determines 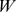), so that 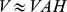. Unlike the previous ones, this NMF variant applies to both nonnegative and mixed-sign data matrices. It allows also the sources in 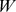 to be of mixed-sign, while the mixing coefficients in 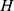 are nonnegative. The factors 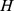 and 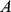 are updated as follows:

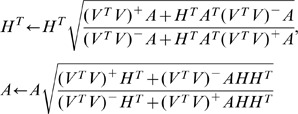
where 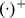 is the positive part of the matrix, where all negative values become zeros; and 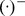 is the negative part of the matrix, where all positive values become zeros.

- We reckon Convex-NMF to be especially well suited to the analysis of MRS data for the following two reasons:
- Firstly, and as just mentioned, because it relaxes the constraints of non-negativity both in the matrix of observed data and in the extracted sources. The analyzed MR spectra include negative peaks, so that we would expect the extracted sources to contain negative values as well. The absolute values of the spectra were used when applying NMF methods other than Convex-NMF. As a result, negative peaks such as the inverted lactate peak are not lost (as they would be if the signal was truncated). In any case, Convex-NMF does not require any *ad hoc* distortion of the observed signal.
- Secondly, because, by restricting 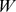 to convex combinations of the columns of 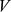 we can, in fact, understand each of the basis or sources as weighted sums of data points (given that 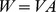). This is a unique feature of Convex-NMF that brings about an interesting result: sources can, to some extent, be considered as cluster centroids or, more abstractly, as *representatives* or *prototypes* of the groupings in which the observed data are naturally structured. As shown in [18], the results of Convex-NMF, if seen as an unsupervised clustering procedure, often agree with those provided by the well-known K-means algorithm [38]. In fact, it is proved in [18] that Convex-NMF is a relaxation of the K-means algorithm. Interestingly, Convex-NMF is bound to generate sparse mixing matrices 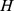
(with many elements taking values close to zero), which are practical as cluster indicators. As a result of all this, the sources obtained by Convex-NMF are likely to be interpretable and similar to data group centroids.

Experiments with the different NMF methods were carried out to identify which of them yielded better results in terms of the correlation between the sources and the average spectra of the tumor and non-tumor areas of each mouse. These average spectra for each mouse were taken from the subsets of voxels labeled as tumor and non-tumor, as described in the previous section.

### NMF Initialization

NMF methods unavoidably converge to local minima. As a result, the extracted NMF bases might be different for different initializations. In this study, six initialization methods were investigated, covering a wide array of approaches: from random initialization, to prototype-based clustering methods such as K-means and Fuzzy C-Means (FCM), which provide a data density-based sample of initial data locations; and to feature extraction techniques such as Principal Component Analysis (PCA), Independent Component Analysis (ICA) and NMF itself, which initialize the algorithm according to the basic eigenstructure of the data.

All the NMF algorithms, for all initializations, were allowed to achieve convergence. Convergence was qualified as the lack of variation in the reconstruction error, from one iteration to the next, over a common set small threshold of value 10^−5^.

### Voxel Labeling using the Mixing Matrix and the Sources

As explained in the introduction, NMF in this study is used as an unsupervised method in the sense that the labels of MRSI voxels are not used to create the data model. The obvious advantage of this approach lies in the fact that the labeling procedure becomes independent of the availability of labeled MRSI datasets. Two further advantages come with this independence: First, the generalization capabilities of the obtained model will not be compromised by the bias introduced by the finite size of the labeled sample. Second, the negative effect of mislabeled cases on the generalization capabilities of the model will be prevented.

In order to represent the data using the source signals obtained through the chosen NMF method, we propose to infer the labels of each voxel only on the basis of the mixing matrix and the calculated source signals. The contribution of each source is estimated by calculating the scalar product between the original voxel spectrum and the reconstructed component of the voxel spectrum that is obtained from one individual source and the corresponding mixing matrix element (i.e. loading) for that voxel. The contribution 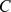 of source 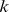 (from 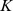 sources) to voxel 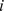 (from 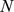 voxels) is then given by:
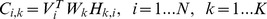
(2)


The predicted label can then be inferred from the values in 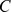 as follows: for each voxel 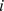, the label 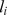 is provided by the source 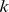 that has the highest value of contribution for that voxel, that is 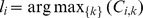.

### Experimental Settings

For each mouse, the data matrix 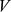 is built with the available MRSI spectra. Its dimension is 692×100 (corresponding to 692 frequencies in the spectral interval of interest, between 0 and 4.5 ppm, and 100 voxels). All the investigated variants of the NMF method, with all considered initialization strategies, were used to calculate the underlying source signals. The interpretation of each source was made by reference to the average spectra of the corresponding mouse brain regions, as previously mentioned. The method was set up to calculate two source signals, under the hypothesis that one of them will represent the tumor, while the other will represent the non-tumor region. This is intended to capture the separation between the two main tissue types.

After calculating the source signals and the mixing matrix, binary (tumor *vs.* non-tumor) labels for each voxel were then generated in a fully unsupervised mode. The correlations between the spectra in each voxel and each source were calculated, and the label was assigned according to the source that yielded the highest correlation. If the correlation between the spectrum of a voxel and both sources was below a threshold of 50%, then we labeled this voxel as ‘undecided’ (thus effectively abstaining from labeling the voxel). Assigning a color for each source, and black for the ‘undecided’ voxels, we created color maps with the labels, that we call “source-based labels maps”.

As an alternative form of representation, we used the contribution values of only the tumor source to each voxel, from Eq.2, obtained as described in the previous section. Color maps were created using these values, with hues of red representing the highest contribution values, which correspond to voxels labeled as tumor, and hues of blue representing the lowest contributions, which correspond to non-tumor. The darkest hue of red corresponds to the highest absolute contribution of the tumor source and darkest blue corresponds to the lowest one. We call these maps “source contributions maps”.

## Results

### A. Source Signals

For illustration, tables 1 and 2 show the correlations obtained between the sources (obtained with the five NMF methods previously described, each with the six different initialization strategies proposed) and the corresponding average spectra of tumor and non-tumor areas, at LTE and STE, respectively, for one of the seven mice, namely C69. The correlation values obtained for the remaining six mice are compiled in tables S1 and S2. Mouse C278 practically does not contain non-tumor area that fulfils the selection criteria outlined in the Material and Methods section. Thus, only a fraction of the voxels was labeled as tumor whereas the rest were labeled as “other”. Therefore, for this mouse in particular, we only made the comparison between the average spectrum of the tumor area and the tumoral source in tables S1 and S2.

**Table 1:** Correlations between the sources and the average spectra for mouse C69 at LTE.

Mouse C69					
init	*euc*	*als*	*alspg*	*alsobs*	*convex*
Random	.976/.940	.977/.938	.975/.928	.976/.939	**.991/.986**
K-means	.975/.941	.977/.938	.976/.939	.976/.939	**.987/.993**
FCM	.975/.940	.977/.938	.976/.939	.976/.939	**.987/.993**
PCA	.934/.879	.977/.938	.976/.938	.976/.939	**.982/.988**
ICA	.969/.800	.977/.938	.976/.938	.976/.939	**.986/.992**
NMF	.976/.938	.977/.938	.976/.939	.976/.939	**.986/.992**

**Figure 2:**
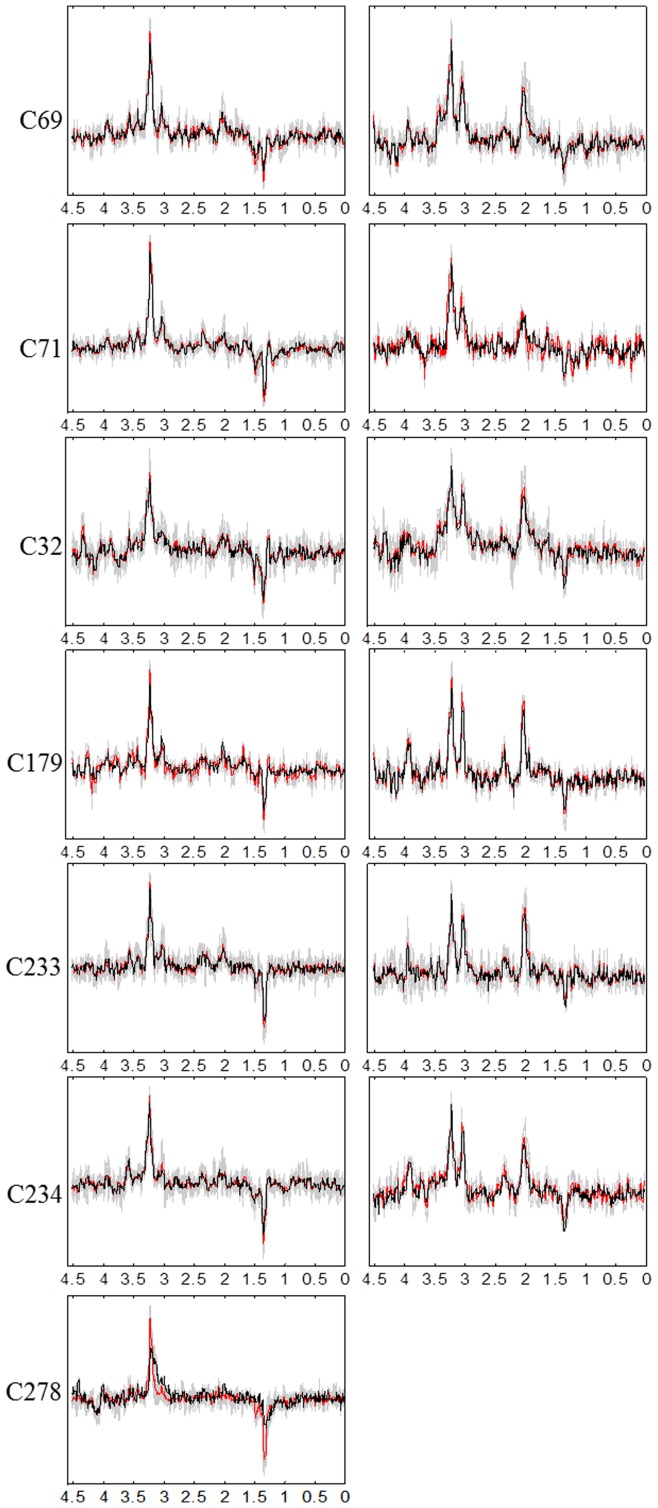
Sources obtained for the seven mice at LTE. The calculated sources are shown in black color, the average spectra in red, and all the labeled spectra in gray. The sources in the left column represent the tumor, and the ones in the right column mainly represent non-tumoral tissue. Frequencies in the horizontal axis are measured in ppm.

**Figure 3:**
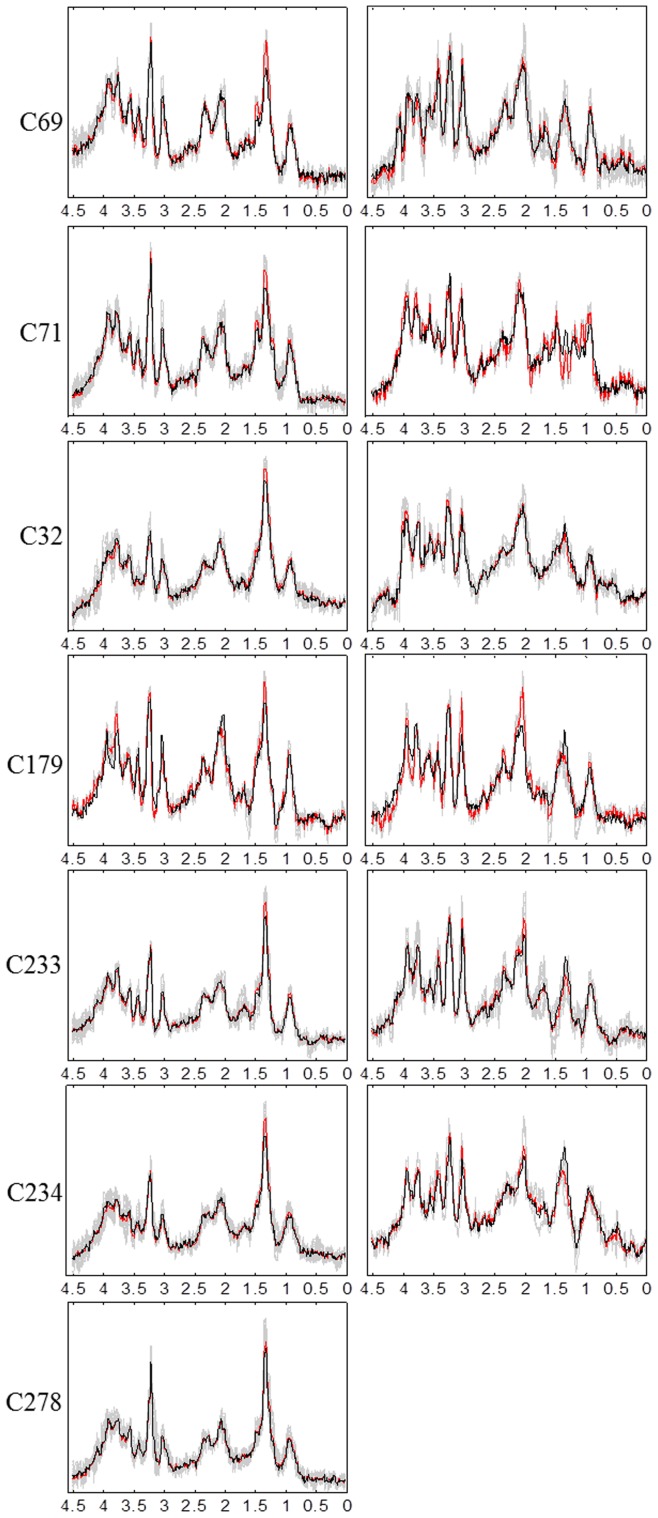
Sources obtained for the seven mice at STE. The calculated sources are shown in black color, the average spectra in red, and all the labeled spectra in gray. The sources in the left column represent the tumor, and the ones in the right column mainly represent non-tumoral tissue. Frequencies in the horizontal axis are measured in ppm.

Table cells should be read as the correlations between the sources and the average spectra (see figures 2 and 3) of the tumor/non-tumor areas. The results of the best performing method, for each initialization condition, are highlighted in bold.

**Table 2:** Correlations between the sources and the average spectra for mouse C69 at STE.

Mouse C69					
init	*euc*	*als*	*alspg*	*alsobs*	*convex*
Random	.957/.989	.967/.991	.902/.990	.967/.991	**.981/.983**
K-means	.968/.990	.967/.991	.968/.991	.968/.991	**.985/.992**
FCM	.965/.990	.967/.991	.968/.991	.968/.991	**.986/.997**
PCA	.915/.811	.967/.991	.969/.991	.968/.991	**.983/.996**
ICA	.809/.990	.967/.991	.962/.991	.967/.991	**.983/.997**
NMF	.968/.991	.967/.991	.968/.991	.968/.991	**.984/.998**

Table cells should be read as in table 1.

Convex-NMF yields, overall and consistently, the best correlation results. The results are also fairly insensitive to the initialization strategy. The source signals calculated in the experiments carried out with all mice at LTE and STE, using Convex-NMF with K-means initialization, are displayed in figures 2 and 3, respectively. In K-means clustering, 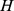 was initialized as 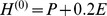, where 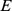 is a matrix of all its elements equal to one, and 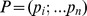 was filled with the cluster indicators, which are based on the cluster indices of each point, such that 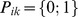, where the ones indicate cluster membership. 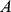 was initialized as 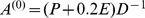, where 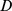 is a diagonal matrix with each element being the number of points in each cluster [18].

In order to visualize the similarities and differences of the calculated sources with respect to the average spectra of areas labeled by the expert as tumor and non-tumor, we superimposed the calculated sources to the average spectra, and to the set of all labeled spectra, as shown in figures 2 and 3. The sources on the left hand side column have a clear pathological profile, as expressed by the presence of high choline, lactate and mobile lipid peaks and low or no NAA [27], [39]. Instead, the sources on the right hand side column mostly represent non-tumor tissue. These results are consistent with some of the Convex-NMF properties described in previous sections: as stated there, the sources obtained by this method are likely to be interpretable and similar to data group centroids, given that each source is a weighted sum of spectra, and given the relation between Convex-NMF and the K-means clustering algorithm.

**Figure 4:**
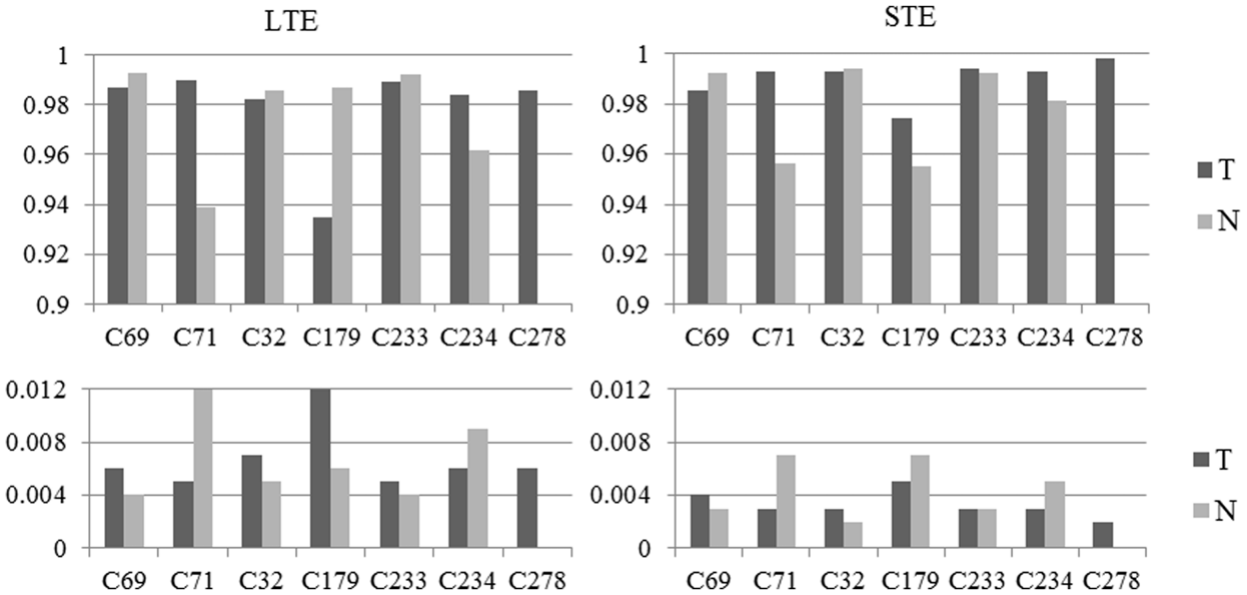
Correlations and errors. Correlations (top row) and errors (bottom row) between the source signals and the average spectra of the labeled areas (as measured by RMSE). Dark gray bars: tumors; light gray bars: non-tumor tissue.

Figure 4 shows detailed correlation (top row) and Root Mean Squared Error (RMSE, bottom row) results for all the analyzed mice at both echo times, where sources were obtained using Convex-NMF with K-means initialization. These results include both tumor (darker bars) and non-tumor tissue (lighter bars).

There is some controversy regarding the uniqueness of the solutions obtained by NMF methods. In order to clarify this for our results, we took one of the studied mice, namely C69 at LTE, and compared the sources obtained starting from 50 random initializations with values in the interval (0,1). Allowing for trivial permutations in the order in which the sources were obtained by the Convex-NMF algorithm, the results were consistently 0.99 or better correlated with the sources. According to this, Convex-NMF is converging to very similar solutions, which from the standpoint of the current study could be considered as they are all one and the same solution.

Secondly, we perturbed the mixing matrix obtained for this class pair, by multiplication with a random matrix with positive elements in the range (0,1). This was used to re-initialize the Convex-NMF algorithm, which converged back to the original solutions, with a very small difference (0.0415±0.02 root mean squared error between the original and the new values). The results of this second test reinforce our previous statement regarding the uniqueness of the solutions obtained by Convex-NMF for the type of data under analysis.

### B. Voxel Labeling and Tumor Delimitation

**Figure 5:**
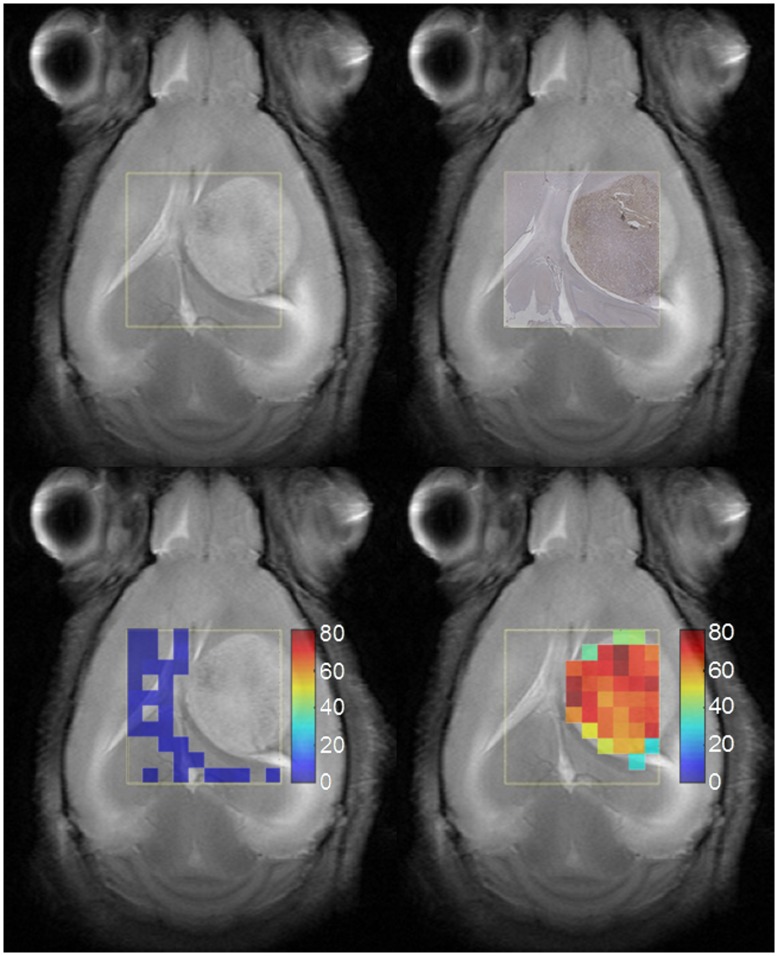
Histology information of mouse C69. T2-W image (top left); Ki67 immunostained digitized slide (top right); Ki67 map with PI≤5% (bottom left); and Ki67 map with PI>30% (bottom right), of mouse C69, bearing a GL261 GBM tumor. The last two maps are superimposed on the corresponding reference T2-W image. Color columns at the bottom show PI scale.

**Figure 6:**
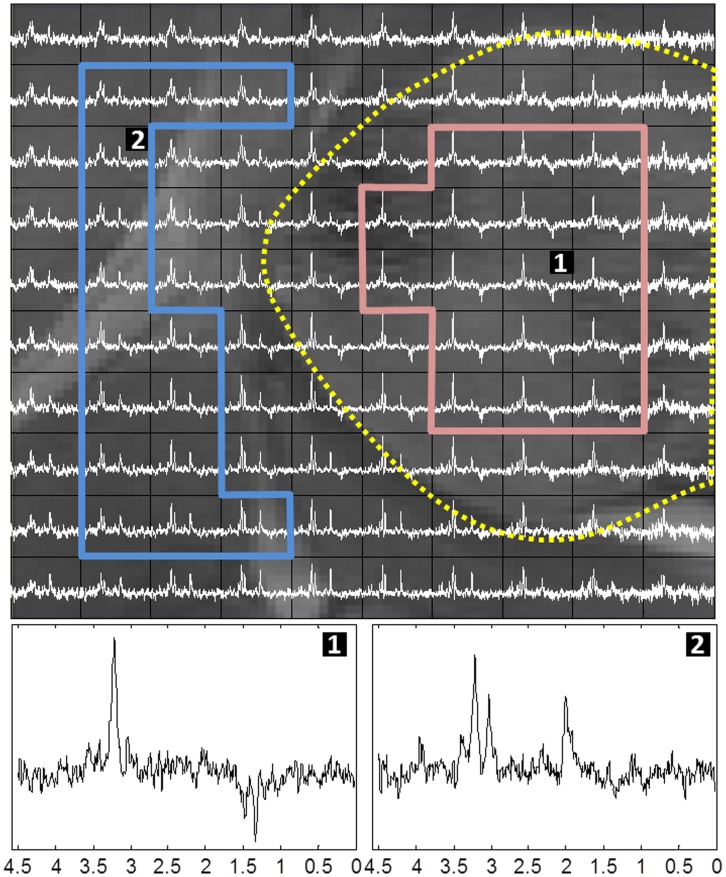
VOI region of mouse C69. T2-W image enlarged and overlaid with the MRSI matrix at LTE; spectra shown in white. The red and blue contours delineate, in turn, characteristic tumor and non-tumor areas used for calculating the average spectra to which the unsupervised sources were compared. The yellow dotted line outlines the tumor as judged from the “anomaly region” on the reference T2-W image. Bottom spectra 1 and 2 arise, enlarged, from the voxels labeled in the top image.

Figure 5 shows the T2-W image for one of the studied mice (C69), together with the respective histology superimposed, using monoclonal antibody against Ki67, and Ki67 PI maps for voxels with PI≤5% and PI>30%. The yellow squares delimit the VOI region. The VOI of the same mouse is further detailed in figure 6, enlarged and overlaid with the 10×10 MRSI spectral matrix at LTE. The areas delimited by red and blue lines correspond to characteristic tumor and non-tumor labels, respectively, labeled as in [32]. These labels will be referred to, later on in the study, as “supervised”. The yellow dotted line in figure 6 delineates the “radiological anomaly region” as judged from the mass shown in the T2-W image.

**Figure 7:**
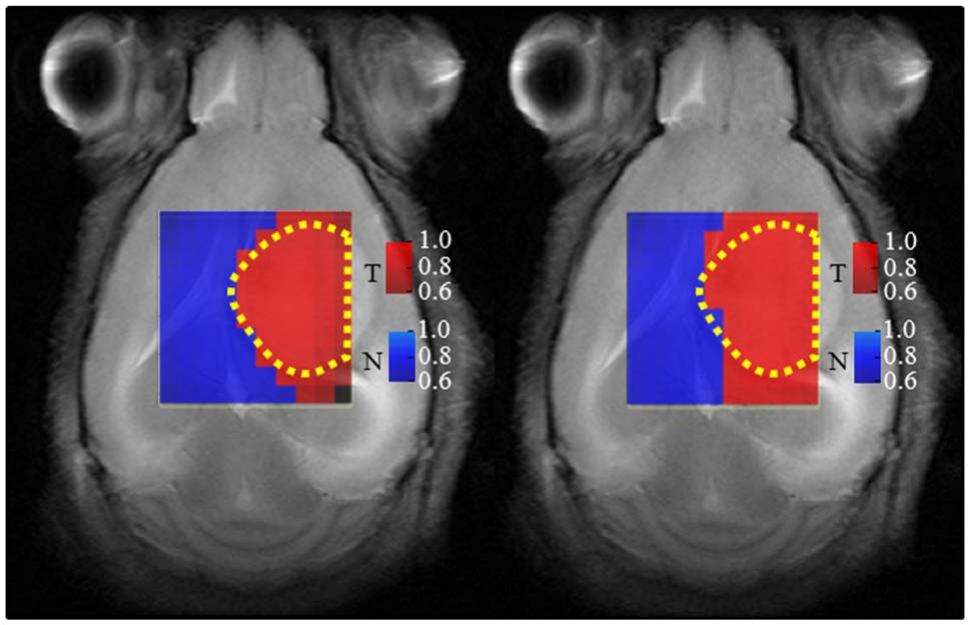
Source-based labels maps generated for mouse C69. The maps obtained at LTE (left) and STE (right) are superimposed to the T2-W reference image. The red color identifies tumor (T), blue identifies non-tumor (N), and black represents ‘undecided’. The color intensities shown in the scale bar on the right hand side correspond to the magnitude of the correlation values between the spectra of the voxels and their representing source. The yellow dotted lines outline the tumor region, as judged from the T2-W image, similar to figure 6.

The source-based labels maps generated for mouse C69 at LTE and STE are shown in figure 7. In this figure we superimposed these maps to T2-W images, to verify the correspondence between the tumor areas described by the sources and the T2-W image.

There is a single black voxel in figure 7 (source-based labels map generated for C69 at LTE). This voxel is labeled as ‘undecided’, indicating that the correlations of the sources with the original spectrum of this voxel (same position in figure 6: poor SNR), are under the 50% threshold.

**Figure 8:**
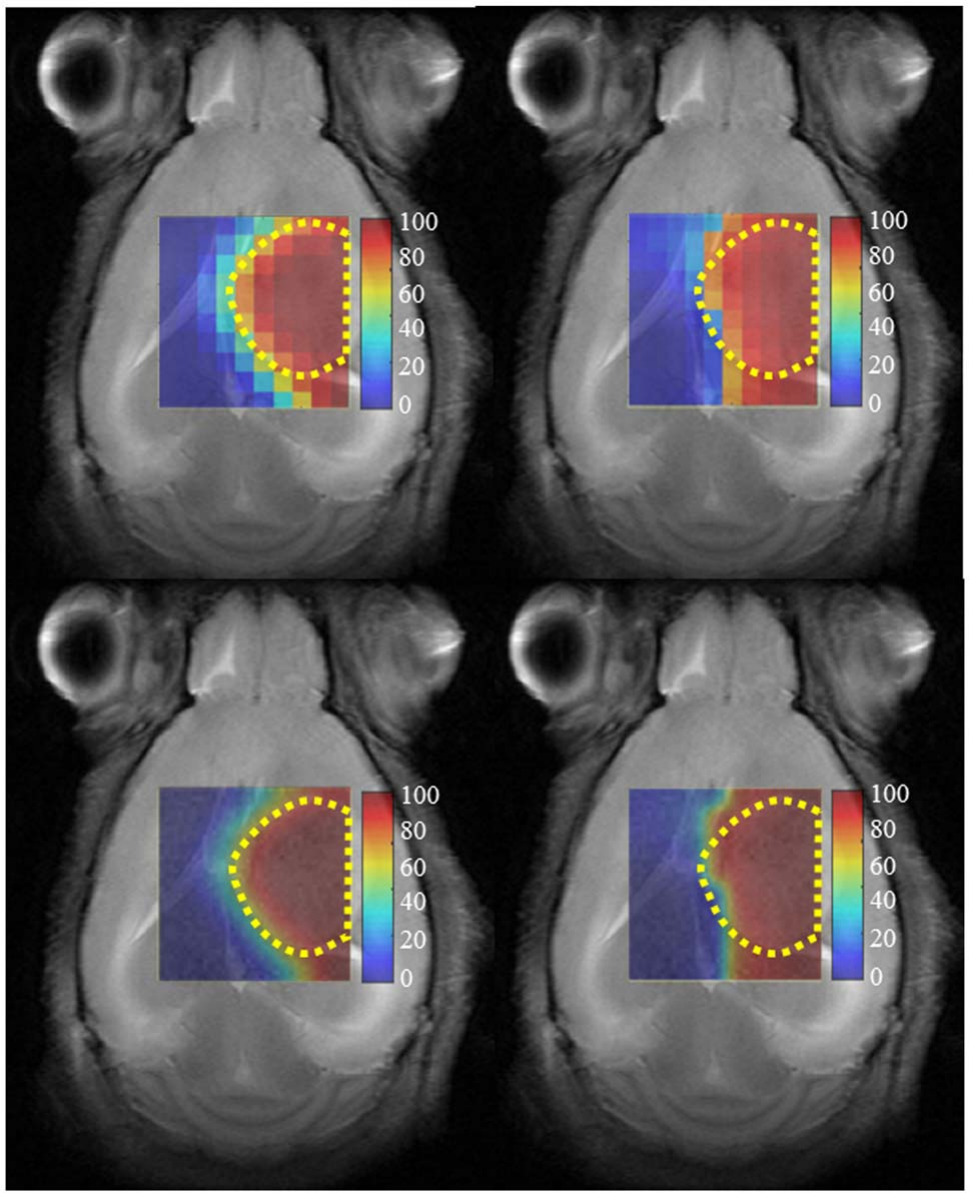
Source contributions maps representing the tumor area of mouse C69. They codify the *C* values of Eq.2 scaled between 0 and 100. The maps obtained at LTE (left column) and STE (right column) are superimposed over the T2-W reference image. The red color identifies the tumor, while blue identifies non-tumoral tissue. The yellow dotted lines outline the tumor region, as judged from the T2-W image, similar to figure 6. Top row: 10×10 grid of voxels. Bottom row: the same map interpolated. Color columns on the right hand side of the maps indicate *C* scale in percentage.

We illustrate the tumor delimitation capabilities of the method proposed in this paper in figure 8. This figure shows the source contributions maps generated for the VOI regions of mouse C69 at LTE and STE. The first row shows the 10×10 grid of voxels, with the corresponding color according to the values of 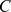 calculated from Eq.2 for the source representing the tumor in each voxel. As in figure 7, this is then superimposed over the T2-W image to verify the high correspondence between the area of the tumor in both images (the source contributions map and the T2-W). The maps displayed in the bottom row are created with a linear interpolation of the maps in the top row, also superimposed over the corresponding T2-W image.

**Figure 9:**
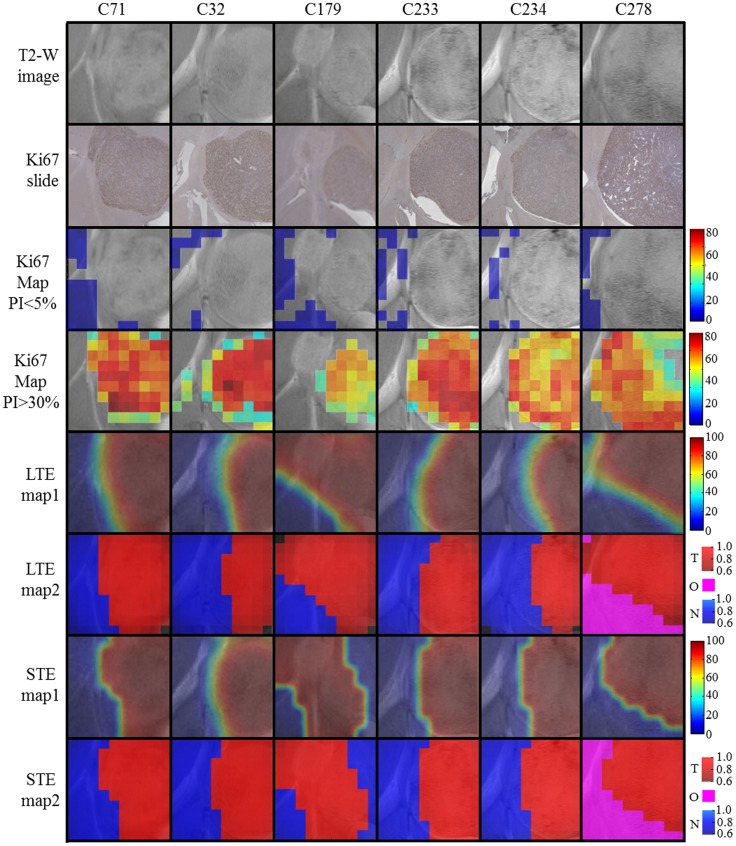
Summary of the results for mice C71, C32, C179, C233, C234, and C278. T2-W images (first row), Ki67 slides (second row), Ki67 maps with PI≤5% (third row), Ki67 maps with PI>30% (fourth row), source contributions maps (interpolated) of the tumoral source calculated at LTE (fifth row), and STE (seventh row), source-based labels maps calculated at LTE (sixth row) and STE (eighth row). Color columns of rows 3 and 4 as in figure 5. Color columns of rows 5 and 7 as in figure 8. Color columns of rows 6 and 8 similar to figure 7, adding a new color (magenta) to represent the region below the main tumor source 50% threshold (O, “other”) in mouse C278. Please note that C278 does not contain non-tumoral tissue voxels that fulfill the labeling criteria for source calculation according to [32], [33].

Figure 9 summarizes the information for the remaining 6 mice (C71, C32, C179, C233, C234, and C278). The first four rows contain the known information about these mice, used here to assess the accuracy of the obtained results, which are compiled in the last four rows. An additional color, magenta, is used in order to represent the area that is not normal brain, but neither is being represented by the main tumoral source (mouse C278, rows 6 and 8).

To determine the extent to which the source-based labels maps were related to the Ki67 maps, we calculated the accuracy of the former as compared to Ki67 PI maps for the tumor region (results for all mice, at LTE and STE, are compiled in table 3). For this, as described in methods, we considered PI>30% to be a safe threshold for the solid tumor region, and PI≤5% as the threshold for non-tumor, our “gold standards” according to the opinion of the expert pathologist. We also calculated the accuracy of the obtained source-based labels maps in relation to the supervised tumor and non-tumor labels, described in [32] and shown in figure 6 for mouse C69. Table 4 contains the results.

**Table 3:** Relationship between the Ki67 maps and the source-based labels maps.

	LTE		STE	
Mouse	T	N	T	N
C69	100(41/41)	93.3(28/30)	97.6(40/41)	86.7(26/30)
C71	91.9(57/62)	91.7(22/24)	83.9(52/62)	91.7(22/24)
C32	66.7(38/57)	100(10/10)	84.2(48/57)	100(10/10)
C179	100(33/33)	66.7(16/24)	66.7(22/33)	37.5(9/24)
C233	86.7(52/60)	100(16/16)	91.7(55/60)	100(16/16)
C234	69.8(44/63)	100(10/10)	87.3(55/63)	100(10/10)
C278	71.1(54/76)	–	71.1(54/76)	–
Mean	83.7	92.0	83.2	86.0

T stands for the accuracy between a PI>30% and the tumor area delineated by the source-based labels maps; while N stands for the accuracy between a PI≤5% and the corresponding non-tumor area. The numbers in parentheses correspond to the number of correctly labeled voxels from the total. The last row contains the mean values for each column.

**Table 4:** Relationship between the supervised labels and the source-based labels maps.

	LTE		STE	
Mouse	T	N	T	N
C69	100(17/17)	100(14/14)	100(17/17)	100(14/14)
C71	100(26/26)	100(7/7)	100(26/26)	100(7/7)
C32	91.7(22/24)	100(10/10)	100(24/24)	100(10/10)
C179	100(9/9)	100(9/9)	88.9(8/9)	66.7(6/9)
C233	100(29/29)	100(12/12)	100(29/29)	100(12/12)
C234	84.4(27/32)	100(5/5)	100(32/32)	100(5/5)
C278	87.5(35/40)	–	92.5(37/40)	–
Mean	94.8	100	97.3	94.5

T stands for the accuracy between the supervised tumor labels (as in figure 6) and the tumor area delineated by the source-based labels maps; while N stands for the accuracy between the non-tumor labels and the corresponding non-tumor area. The numbers in parentheses correspond to the number of correctly labeled voxels from the total. The last row contains the mean values for each column.

Table 5 compiles the sensitivity (true positive rate, 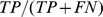, where true positive (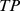) cases are tumor voxels correctly labeled as tumors, and false negative (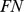) cases are non-tumor voxels labeled as tumors) and specificity true negative rate, 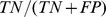, where true negative (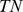) cases are non-tumor voxels correctly identified as non-tumors, and false positive (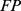) cases are tumor voxels labeled as non-tumors) of the obtained source-based labels maps.

**Table 5:** Sensitivity and specificity for the source-based labels maps.

	LTE		STE	
Mouse	Ki67	Supervised	Ki67	Supervised
C69	1.00/0.93	1.00/1.00	0.98/0.87	1.00/1.00
C71	0.92/0.92	1.00/1.00	0.84/0.92	1.00/1.00
C32	0.67/0.46	0.92/1.00	0.84/1.00	1.00/1.00
C179	1.00/0.50	1.00/1.00	0.67/0.38	0.89/0.86
C233	0.87/0.48	1.00/1.00	0.92/1.00	1.00/1.00
C234	0.70/0.47	0.84/1.00	0.87/1.00	1.00/1.00
C278	0.71/−	0.88/−	0.71/−	0.93/−
Mean	0.84/0.63	0.95/1.0	0.83/0.86	0.97/0.98

Sensitivity and specificity (sensitivity/specificity) calculated for the source-based labels maps with respect to the Ki67 maps (columns named as Ki67, computed as in table 3), and the supervised labels provided (columns named as ‘Supervised’, computed as in table 4), for each mouse, at LTE and STE. The last row contains the mean values for each column.

In most cases the tumor sources overlapped relatively well the PI>30% (table 5 and figures 7, and 9), but there were some exceptions. In mouse C69, there was a spread of the tumor source outside the mass as seen on MRI towards the caudal part of the brain. In mouse C179 the source spread towards the smaller mass, which had been labeled as less than 30% PI by the Definiens coregistration.

**Figure 10:**
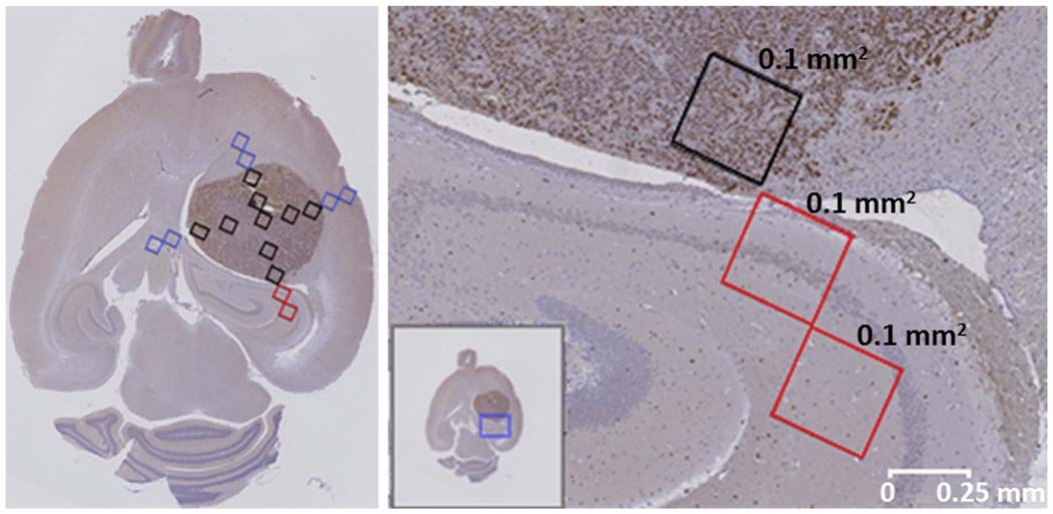
Ki67 preparation from mouse C69. On the left hand side, 0.1 mm^2^ manual sampling areas (colored rectangles) for positive Ki67 cells’ evaluation (brown spots): black squares, sampling inside the tumor mass; blue squares, samplings adjacent to tumor mass; red squares, *Cornu Ammonis* samplings adjacent to tumor mass, shown enlarged on the right. On the right hand side, rectangles from top to bottom: PI was 57, 13 and 13.3%; the first square was inside the tumor mass, the second one was adjacent to it, and the third one was outside the tumor. Cellularity was 5100, 1200 and 750 cells/mm^2^, respectively. Non-proliferating nuclei were stained in blue and proliferating nuclei in brown. The small insert in the right image (blue square) shows the location of the enlarged image with respect to the whole brain. The white bar at the bottom-right shows scale.

**Figure 11:**
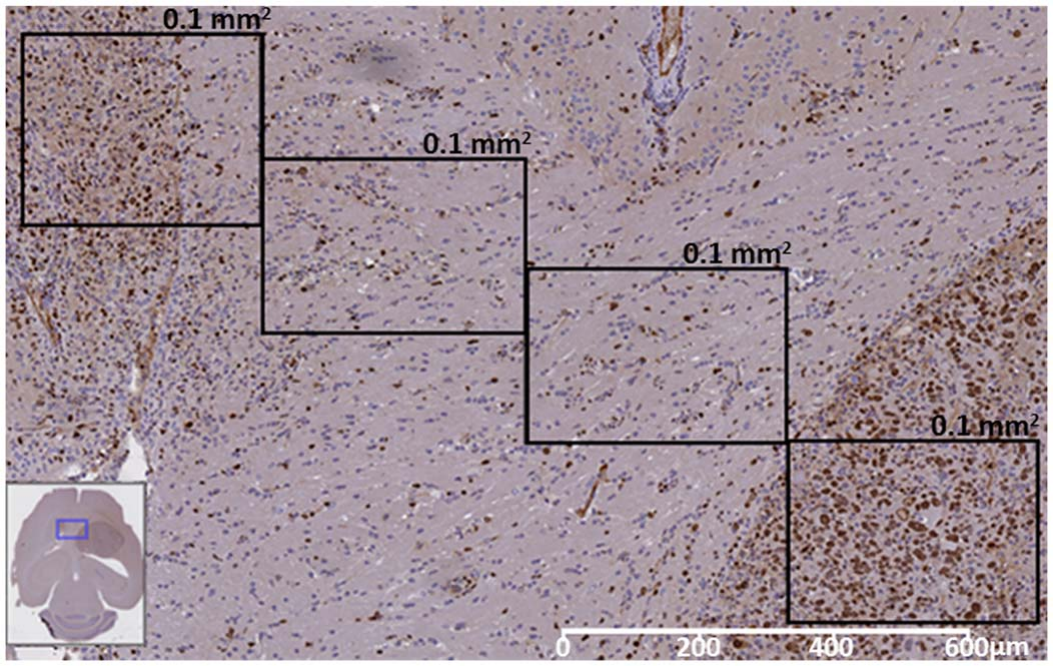
Ki67 preparation from mouse C179. The connecting region between the two tumor masses is shown enlarged, displaying three 0.1 mm^2^ areas manually sampled. From top (smaller mass) to bottom (main mass), PI: 40%, 21.5%, 24.5%, and 62.7%. White bar at bottom-left corner shows the scale. The insert at the bottom left corner shows the location of the enlarged image (blue rectangle) with respect to the whole brain.

In both cases, an additional manual sampling and count of 0.1 mm^2^ squares along the vertical and horizontal axes of the tumor (figures 10 and 11) revealed that:

In mouse C69 the tumor was well delimited, lacking surrounding infiltrative areas. However, the bottom (caudal) edge of the tumor, presented an area with a PI of 13% due to proliferating glial cells in the *Cornu Ammonis* (CA) of the hippocampus. Despite this, the top (rostral), right and left immediately adjacent to the tumor areas had a PI of 0, 5.4, and 3.3% respectively, consistent with a well delimited tumor mass and the <5% threshold for non-tumor set before. These results may explain the spread of the source of the tumoral area observed outside the caudal T2-W anomaly region (figure 7). Additionally, the mean of samplings performed inside the tumor mass yielded a PI of 73.9% and cellularity of 3647 cells/mm^2^ (2251 proliferating cells/mm^2^), consistent with a similar study (ca. 2000 proliferating cells/mm^2^), reported in [40].

Regarding mouse C179 in which two masses are evident on the MRI (figure 9) both T2-W and source based maps recognize tumor better than our self-imposed “gold standard” (PI>30%). In this respect, figure 11 shows a transition area of proliferating cells, between the big and the small mass. The two samplings in the transition area showed PI values of 24.5 and 21.5% and the third sampling, already in the smaller mass, had a PI of 40%. These values are consistent with the >30% PI for solid tumor and above the 5% PI for normal brain, and agree both with the T2-W and the source maps at both echo times. A possible explanation for this disagreement between the values provided by the automated coregistration method and the MRI and the MRSI can be that an affine was applied instead of an elastic transformation.

**Figure 12:**
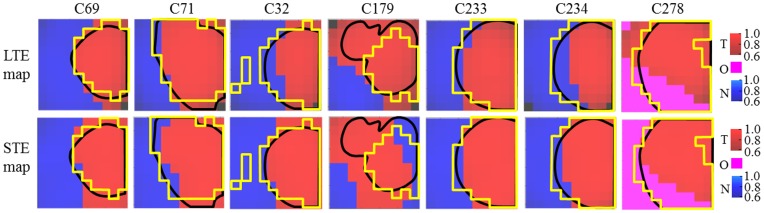
Delineation of the tumor area superimposed to the source-based labels maps. Delineation of the tumor area as suggested by the MRI-abnormal region (black solid line) and the PI>30% regions (yellow solid line), superimposed to the source-based labels maps for the seven mice studied. Red region: tumor, blue region: non-tumor, magenta region: “other”.

It should also be kept in mind that the size of the MRSI coded pixels (0.55×0.55 mm in plane resolution and 1 mm slice thickness in the 3^rd^ dimension) [27] leads to partial volume effects, causing most borderline pixels to contain PI contributions from both tumor and non-tumor regions and artificially increasing (or decreasing) the average PI. These may explain as well why, particularly in mice C32 and C234, the source maps are smaller than the T2-W image (figure 12). Histopathology slides for these two mice were also manually checked and for example, in mouse C32, the PI for the periphery was comprised in the 58.5–76.9% range.

No accuracy/sensitivity/specificity calculation has been performed for the borderline pixels or for Ki67 regions between 5 and 30% Ki67 because of the confluence of several problems. Namely, 1) restricted MRSI resolution (0.55×0.55×1 mm) compared with the MRI resolution (0.15×0.15×1 mm), 2) imperfect registration of *in vivo* and *ex vivo* histopathology data, and 3) self-imposed restriction of the number of sources to be used to two in this initial discrimination study of tumor and non-tumor regions. Still, we have attempted an approximation to a full account of the acquired VOI with respect to its tumor/non-tumor recognition, and for this the gold standard threshold for tumor has been maintained at >30% while all regions with PI below 30% have been combined into the “non-tumor” label. This has produced the results shown in figure 12 and table 6.

An interesting result is that comparison with Ki67 maps yields in all cases lower sensitivity and specificity (table 6) than comparison with the supervised labels (table 5). This also happens when correlation is calculated (table 3 *vs.* table 4). This is probably caused by the already mentioned effect of taking all voxels (including borderline pixels) in the calculation when analyzing images, while the supervised labeled images do not take into account borderline pixels between tumor and non-tumor tissue, as well as the specific coregistration algorithm used.

**Table 6:** Sensitivity and specificity for tumor delimitation using PI≤30% as non-tumor.

		Source-based labels maps	
Mouse	T2-W	LTE	STE
C69	1.00/0.85	1.00/0.86	0.98/0.76
C71	1.00/0.74	0.92/0.81	0.84/0.79
C32	0.93/0.77	0.68/0.88	0.82/0.86
C179	0.97/0.55	1.00/0.38	0.67/0.31
C233	1.00/0.90	0.87/0.90	0.90/0.95
C234	1.00/0.97	0.71/1.00	0.87/0.97
C278	1.00/0.71	0.71/0.54	0.70/0.83
Mean	0.99/0.78	0.84/0.77	0.83/0.78

Sensitivity and specificity (sensitivity/specificity) calculated with respect to the delineation provided by the T2-W image, and also with respect to the source-based labels maps at LTE and STE, for each mouse. The “gold standard” used here is the Ki67 maps. PI>30% represents tumor, while PI≤30% is now taken to represent non-tumor. The last row contains the mean values for each column.

The borderline regions of the tumors according to the T2-W image are heterogeneous and contain regions of anomaly which the source maps incorporate into either tumor (mostly in mice C69, C179 and C278) or non-tumor regions (mostly in mice C32, C233 and C234), depending of the mouse analyzed, thus providing a different delimitation of the abnormal mass which may be relevant for its evaluation and follow-up.

## Discussion

### A. Source Signals

The results reported in tables 1, 2, S1 and S2 indicate that NMF methods are capable of extracting tissue type-specific (tumor or non-tumor) sources. In terms of correlation, Convex-NMF outperforms the other methods tested, with small differences for different initialization strategies. The higher correlations provided by Convex-NMF are very noticeable for non-tumor tissue, especially at LTE, for most mice. In some cases, the initialization results yielded by PCA and ICA are very good, e.g. C71 (at LTE and STE) and C32 (at LTE), but very poor in others, e.g. C179 (at LTE), becoming much worse than random. K-means and FCM initializations yielded very similar results, except in some cases in which FCM performed worse, e.g. C179 (at LTE and STE). The combination of Convex-NMF and K-means initialization was the most stable of all, providing very good correlation results.

In a previous study [41], where we assessed the abilities of two variants of ICA [42], [43], namely *JADE* [44] and *FastICA* [45], to identify the constituent tissue types in single-voxel MRS, ICA showed no advantages over NMF methods.

With respect to the acquisition conditions, both TE sources seem to perform similarly in average (tables 3, 4, 5, 6) for delineating tumor and normal tissue, with quantitative variations depending on the specific mouse studied (table 6 and figure 12), but see next section for further comments on this.

The results in figures 2 and 3 support our initial assumption that the two main sources obtained by Convex-NMF correspond to tumor and non-tumor tissues.

GBM are highly malignant, WHO grade IV tumors [46], usually showing a strong mobile lipid MRS signal (at *ca*. 1.3ppm), most evident at STE [47] (see figure 3, left column). Normal brain tissue is usually characterized by a clear NAA peak at 2.02ppm with similar height to creatine (3.03ppm) and choline-containing compound (3.21 ppm) peaks in mice models [39]. In the tumor area, the creatine peak height decreases, while the total choline peak increases. Lactate (1.3ppm) resonances appear inverted at LTE (see figure 2, left column) but overlap lipid signals at STE (see figure 3, left column).

### B. Voxel Labeling and Tumor Delimitation

The red area obtained for both source-based labels maps in figure 7 corresponds to the tumoral area previously delimited and to the true proliferative tumor area, as shown by the Ki67 images (figure 5). This similarity has been achieved using our proposed method to generate source-based labels maps in a fully unsupervised way. Previous approaches for brain tumor segmentation based on MRS labeling, such as [48], are all supervised. This unsupervised approach would allow us to provide a labeling prediction independent of the availability of a labeled data set.

The source-based labels maps have been the type of maps chosen for detailed quantitative evaluation of the outcome of the proposed approach (tables 3, 4, 5, 6 and figure 12). Still, the use of the source contributions maps (figures 8, 9) may have advantages in future studies to better sample tissue transition zones or when more than two sources need to be considered.

While most of the existing unsupervised strategies are based on MRI, the area of metabolic abnormality for human brain tumors is usually larger than the area delimited by conventional MRI [49]. This may lead, e.g., to inaccuracies in the evaluation of therapy response volumes.

Previous work [23] applying NMF to MRSI data of pathological states, either on acute or chronic phases, yielded a recovered abnormal image beyond the regions of abnormal signal intensity on T2-W Fluid Attenuated Inversion Recovery (FLAIR) images (T2-W with flowing liquid attenuation) [50], whose match was evaluated only visually. While correctly mapping the abnormalities, our approach provides a good delineation of the abnormal area and quantitative estimates of the prediction accuracy. In addition, since we applied Convex-NMF to a preclinical model rather than to human patients, there is an authentic biological correlate of the true proliferative tumor area, as shown by the histopathology co-localized images (figures 5, 9 and 12). Our results match well the tumoral area (figure 12 and table 6), with the limitations already pointed out in the results section and discussed below.

Despite a particular mouse (C179), the remaining six mice studied showed a clearly circumscribed tumoral mass, essentially with minor or no infiltration outside the tumor. Mouse C69, already discussed, had a tumoral source that extended over MRI-normal areas of the brain in its caudal part (figure 10), which corresponded to PI = 13%. In fact, Ki67 positive cells detected outside the tumor mass in this caudal part were not tumoral, but glial cells (morphologically identified by the pathologist). In this respect, it has been described that proliferative reactive astrocytes (astrogliosis) are attracted to brain tumors [51]. Anatomically, this was observed in the hippocampus region, which is also known to contain proliferating neural stem cells (NSC) [52]. Indeed, increased proliferation of NSC and tropism towards GL261 GBM has been demonstrated [53]. A combination of these facts may explain that source analysis recognized this as abnormal region (tumor plus astrogliosis).

Concerning the non-tumor area of mouse C179, data shown in figures 9, 11 and 12, and in tables 3 and 4, suggest that the sub-optimal agreement of source-derived maps (especially at STE) and Ki67 maps may have its origin in the existence of two masses, instead of just one. This will produce a higher percentage of borderline voxels and, accordingly, a higher partial volume effect. Furthermore, in this particular instance, both T2-W and source based maps recognize tumor better than our self-imposed “gold standard” (PI>30%). This may be caused in this case by labeling “dilution” when considering the decreased MRSI-like resolution used in the Ki67 grid. In this respect, figure 11, shows a borderline area for the C179 small mass which did already provide a manual PI count (40%) clearly above the tumor “threshold”, also in agreement with the T2-W and source map result.

Furthermore, in mouse C278 the source for tumor delimits a clearly smaller area than the abnormal brain mass. This was because in this case lacking a proper normal brain area two sources were sought and the results on tables 3, 4, 5, 6, are calculated only according to one of the sources (the most correlated with tumor). In this case, the appropriate could have been to combine the regions produced by the two sources, but we decided to only keep the most correlated one, as for the other six mice, for calculations. Finally, when comparing the T2-W images with the Ki67 maps and the source-based labels maps with the Ki67 maps (table 6 and figure 12), sensitivity was higher for T2-W than for the source maps (particularly due to mice C32, C233, C234 and C278 for the above-discussed reasons). In contrast, specificity was similar for the T2-W area and the source-based labels maps. In mouse C179 the metabolic abnormality area is clearly underestimated by the Ki67 map, which has already been discussed, and it is for this reason that specificity is so low.

When borderline pixels are considered we must recognize that there are some mismatches, both for the T2-W images and for the maps of the sources with respect to the “gold standard” PI map from histopathology. In some cases the source-based map covers a smaller region than the “gold standard” while in other cases it covers a larger area. The MRI-based T2-W image provides an overall similar rendering to the source map when recognizing non-tumor (specificity average values 0.78 *vs.* 0.77 and 0.78) while being slightly better when categorizing tumor (sensitivity average values 0.99 *vs.* 0.84 and 0.83). Still, these mismatches have to be taken with due perspective. In short, the metabolomic map provided by the sources may be seeing in some cases different information than the other T2-W *in vivo* map. The major reason for this slight underestimation of the abnormal region in some cases, specifically C32 and C234 al LTE, may be the restricted resolution of MRSI as compared to MRI data. On the other hand, recognition of metabolomic abnormality seems better using sources for C69, C179 and C278, but for different reasons in each case (caudal tail non-tumoral cells proliferation, PI close to threshold, VOI too small for the tumor size).

A possible criticism to our approach is the limitation of the automated Ki67 maps, and the need to perform additional manual counts to counter-check for discrepant PI values with respect to the sources obtained. However, coregistration of whole mouse brain with MRI/MRSI is not a straightforward task, first of all because the minor deformation suffered by the sample upon dehydration and the paraffin embedding processes and second for the lack of available software. Possible future improvements in the coregistration could imply using elastic transformations, but at the time when we started this study, we could not foresee this problem which we overcome resorting to manual cell counts based in anatomical references. Note as well that our histology sections were 3 µm thick while the MRSI VOI slice width was 1 mm, and the diameter of a tumor cell is around 10–20 µm, hence another complicating factor. The specific histology slide was chosen by the pathologist based again on pure anatomical criteria.

Other, perhaps simpler, approaches would also be feasible for brain tumors, such as the CNI index (essentially a choline/NAA ratio normalized to the normal contralateral brain ratio). In [16], cancer was predicted with 86% specificity and 90% sensitivity using a CNI threshold of 2.5, but, due to technical and ethical limitations, only selected target regions obtained by image-guided surgery could be evaluated by comparison with the histopathology gold standard - this was not the case in our preclinical study. In our case specificity, when considering selected target regions (supervised labels), was 100% for LTE maps; sensitivity was 100% for 4/7 mice and between 84–92% in the remaining 3/7 mice (table 5). It may also be worthwhile to point out here that the CNI index may also show miss-registration with respect to the abnormal mass region according to MRI. Thus, work by Nelson et al. [54] did show a much smaller “metabolic lesion” (with a CNI threshold of 3) area than the overall T2-W “anomaly region” area in a Glioblastoma patient. Finally, the hyperpolarized lactate/pyruvate ratio may also provide a handle for tumor/brain discrimination, both in developing tumor and upon therapy response [55], [56], although the low lactate signal detectable in normal brain tissue and MRSI resolution issues may also cause difficulties in defining the border between normal and abnormal tissue.

### Conclusions

The clinical management of human brain tumors is an important and delicate challenge for radiologists, who have to make their decisions on the basis of indirect evidence gathered through non-invasive techniques.

One of the main sources of uncertainty in this context arises from the difficulty of appropriately delimiting the pathological area of the brain. In this study, we have provided evidence supporting that a robust delimitation can be achieved through the application of blind source extraction techniques to ^1^H-MRS multi-voxel data. More specifically, we have proposed and tested a fully unsupervised methodology that uses Convex-NMF source extraction to consistently delimitate the tumor region. This method is successfully benchmarked against alternative NMF methodologies. The accuracy of tissue delineation was quantified by comparison with the gold standard of tissue assignment, by direct histopathological measurements in the tumoral region.

## Supporting Information

Table S1 Correlations between the sources and the average spectra for the remaining mice at LTE.
(DOC)

Table S2 Correlations between the sources and the average spectra for the remaining mice at STE.
(DOC)
