# Supplementary material for: Convex Non-Negative Matrix Factorization for Brain Tumor Delimitation from MRSI Data
Source: PLoS One. 2012 Oct 23;7(10):e47824. doi: 10.1371/journal.pone.0047824 (PMC3479143; doi:10.1371/journal.pone.0047824)
Supplement: Table S1 — Correlations between the sources and the average spectra for the remaining mice at LTE. (DOC) [file pone.0047824.s001.doc]

**Table S1.** Correlations between the sources and the average spectra for the remaining mice at LTE.

| **Mouse C71** | | | | | |
| --- | --- | --- | --- | --- | --- |
| init | *euc* | *als* | *alspg* | *alsobs* | *convex* |
| Random | .987/.910 | .990/.906 | .988/.914 | .990/.906 | **.988/.923** |
| K-means | .989/.906 | .990/.906 | .990/.906 | .990/.906 | **.990/.939** |
| FCM | .987/.908 | .990/.906 | .990/.906 | .990/.906 | **.993/.936** |
| PCA | .893/.870 | .990/.906 | .990/.906 | .990/.906 | **.992/.946** |
| ICA | .989/.739 | .990/.906 | .990/.905 | .990/.906 | **.992/.944** |
| NMF | .990/.906 | .990/.906 | .990/.906 | .990/.906 | **.991/.980** |
| **Mouse C32** | | | | | |
| init | *euc* | *als* | *alspg* | *alsobs* | *convex* |
| Random | .947/.952 | .942/.961 | .940/.955 | .941/.961 | **.987/.990** |
| K-means | .941/.959 | .942/.961 | .941/.961 | .941/.961 | **.982/.986** |
| FCM | .941/.958 | .942/.961 | .941/.961 | .941/.961 | **.982/.989** |
| PCA | .932/.786 | .942/.961 | .942/.961 | .941/.961 | **.989/.990** |
| ICA | .951/.905 | .942/.961 | .942/.961 | .941/.961 | **.988/.990** |
| NMF | .941/.961 | .942/.961 | .941/.961 | .941/.961 | **.984/.989** |
| **Mouse C179** | | | | | |
| init | *euc* | *als* | *alspg* | *alsobs* | *convex* |
| Random | .820/.883 | .842/.894 | .834/.881 | .841/.894 | **.904/.895** |
| K-means | .828/.888 | .842/.894 | .841/.894 | .841/.894 | **.935/.987** |
| FCM | .821/.882 | .842/.894 | .841/.894 | .841/.894 | **.875/.894** |
| PCA | **.910/.866** | .842/.894 | .841/.894 | .841/.894 | .928/.854 |
| ICA | .925/.773 | .842/.894 | .837/.892 | .841/.894 | **.927/.856** |
| NMF | .841/.894 | .842/.894 | .841/.894 | .841/.894 | **.890/.908** |
| **Mouse C233** | | | | | |
| init | *euc* | *als* | *alspg* | *alsobs* | *convex* |
| Random | .958/.958 | .962/.958 | .954/.961 | .962/.958 | **.988/.994** |
| K-means | .959/.958 | .962/.958 | .962/.958 | .962/.958 | **.989/.994** |
| FCM | .959/.957 | .962/.958 | .962/.958 | .962/.958 | **.989/.994** |
| PCA | .960/.714 | .962/.958 | .962/.958 | .962/.958 | **.990/.996** |
| ICA | .967/.931 | .962/.958 | .962/.958 | .962/.958 | **.989/.996** |
| NMF | .962/.958 | .962/.958 | .962/.958 | .962/.958 | **.989/.995** |
| **Mouse C234** | | | | | |
| init | *euc* | *als* | *alspg* | *alsobs* | *convex* |
| Random | .963/.914 | .963/.923 | .958/.926 | .963/.923 | **.983/.962** |
| K-means | .963/.918 | .963/.923 | .963/.923 | .963/.923 | **.984/.962** |
| FCM | .962/.916 | .963/.923 | .963/.923 | .963/.923 | **.983/.965** |
| PCA | .968/.726 | .963/.923 | .963/.922 | .963/.923 | **.987/.967** |
| ICA | .973/.751 | .963/.923 | .963/.922 | .963/.923 | **.985/.966** |
| NMF | .963/.923 | .963/.923 | .963/.923 | .963/.923 | **.983/.965** |
| **Mouse C278** | | | | | |
| init | *euc* | *als* | *alspg* | *alsobs* | *convex* |
| Random | .964/- | .964/- | .964/- | .964/- | **.980/-** |
| K-means | .964/- | .964/- | .964/- | .964/- | **.986/-** |
| FCM | .964/- | .964/- | .964/- | .964/- | **.984/-** |
| PCA | .864/- | .964/- | .964/- | .964/- | **.978/-** |
| ICA | .965/- | .964/- | .964/- | .964/- | **.983/-** |
| NMF | .964/- | .964/- | .964/- | .964/- | **.983/-** |

Table cells should be read as in table 1 of the manuscript.
