# Supplementary material for: Convex Non-Negative Matrix Factorization for Brain Tumor Delimitation from MRSI Data
Source: PLoS One. 2012 Oct 23;7(10):e47824. doi: 10.1371/journal.pone.0047824 (PMC3479143; doi:10.1371/journal.pone.0047824)
Supplement: Table S2 — Correlations between the sources and the average spectra for the remaining mice at STE. (DOC) [file pone.0047824.s002.doc]

**Table S2.** Correlations between the sources and the average spectra for the remaining mice at STE.

| **Mouse C71** | | | | | |
| --- | --- | --- | --- | --- | --- |
| init | *euc* | *als* | *alspg* | *alsobs* | *convex* |
| Random | .966/.969 | **.982/.975** | .951/.958 | .981/.975 | .975/.874 |
| K-means | .986/.972 | **.989/.975** | **.989/.975** | **.989/.975** | .993/.956 |
| FCM | .982/.973 | **.989/.975** | **.989/.975** | **.989/.975** | **.994/.968** |
| PCA | .937/.723 | .982/.975 | .989/.969 | .989/.975 | **.993/.983** |
| ICA | .837/.966 | .982/.975 | .977/.975 | .981/.975 | **.993/.970** |
| NMF | .982/.975 | .984/.975 | .984/.975 | .984/.975 | **.994/.974** |
| **Mouse C32** | | | | | |
| init | *euc* | *als* | *alspg* | *alsobs* | *convex* |
| Random | .938/.950 | .939/.980 | .905/.925 | .943/.981 | **.984/.963** |
| K-means | .987/.986 | .989/.992 | .989/.992 | .989/.992 | **.993/.994** |
| FCM | .985/.984 | .989/.992 | .989/.992 | .989/.992 | **.995/.996** |
| PCA | .937/.782 | .989/.980 | .990/.955 | .990/.977 | **.994/.998** |
| ICA | .854/.977 | .939/.983 | .933/.988 | .939/.991 | **.995/.996** |
| NMF | .940/.981 | .943/.984 | .944/.984 | .946/.985 | **.995/.997** |
| **Mouse C179** | | | | | |
| init | *euc* | *als* | *alspg* | *alsobs* | *convex* |
| Random | .865/.910 | .881/.906 | .781/.690 | .888/.911 | **.975/.955** |
| K-means | .925/.887 | .890/.913 | .891/.914 | .891/.911 | **.974/.955** |
| FCM | .888/.936 | .890/.913 | .891/.911 | .891/.911 | **.958/.936** |
| PCA | .921/.922 | .890/.912 | .899/.916 | .893/.916 | **.971/.941** |
| ICA | .819/.847 | .886/.909 | .892/.914 | .892/.915 | **.971/.941** |
| NMF | .884/.908 | .890/.912 | .891/.913 | .891/.913 | **.962/.926** |
| **Mouse C233** | | | | | |
| init | *euc* | *als* | *alspg* | *alsobs* | *convex* |
| Random | **.962/.980** | .939/.981 | .900/.965 | .942/.985 | .976/.951 |
| K-means | .989/.982 | .990/.986 | .990/.986 | .990/.986 | **.994/.992** |
| FCM | .987/.983 | .990/.986 | .990/.986 | .990/.986 | **.997/.997** |
| PCA | .935/.786 | .990/.981 | .990/.970 | .990/.979 | **.997/.996** |
| ICA | .892/.958 | .939/.985 | .926/.987 | .935/.987 | **.997/.997** |
| NMF | .941/.981 | .950/.986 | .948/.985 | .952/.987 | **.997/.997** |
| **Mouse C234** | | | | | |
| init | *euc* | *als* | *alspg* | *alsobs* | *convex* |
| Random | .957/.939 | .979/.952 | .922/.941 | **.980/.964** | .962/.913 |
| K-means | .986/.963 | .989/.972 | .989/.972 | .989/.972 | **.993/.981** |
| FCM | .982/.963 | .989/.972 | .989/.972 | .989/.972 | **.995/.986** |
| PCA | .936/.782 | .983/.952 | .990/.934 | .990/.943 | **.995/.989** |
| ICA | .901/.938 | .979/.955 | .972/.969 | .979/.972 | **.994/.987** |
| NMF | .979/.954 | .983/.969 | .983/.968 | .985/.970 | **.995/.989** |
| **Mouse C278** | | | | | |
| init | *euc* | *als* | *alspg* | *alsobs* | *convex* |
| Random | .957/- | .975/- | .896/- | .980/- | **.988/-** |
| K-means | .987/- | .985/- | .985/- | .985/- | **.998/-** |
| FCM | .981/- | .985/- | .985/- | .985/- | **.996/-** |
| PCA | .865/- | .980/- | .972/- | .976/- | **.992/-** |
| ICA | .897/- | .980/- | .972/- | .977/- | **.995/-** |
| NMF | .981/- | .985/- | .984/- | .985/- | **.994/-** |

Table cells should be read as in table 1 of the manuscript.
